# Fruit and vegetable intake and incident and persistent poor sleep quality in a rural ageing population in South Africa: longitudinal study

**DOI:** 10.1192/bjo.2022.548

**Published:** 2022-07-28

**Authors:** Supa Pengpid, Karl Peltzer

**Affiliations:** Department of Health Education and Behavioral Sciences, Faculty of Public Health, Mahidol University, Bangkok, Thailand; and Department of Public Health, Sefako Makgatho Health Sciences University, Pretoria, South Africa; Department of Psychology, University of the Free State, Bloemfontein, South Africa; and Department of Psychology, College of Medical and Health Sciences, Asia University, Taichung, Taiwan

**Keywords:** Fruit and vegetable intake, sleep quality, longitudinal study, South Africa

## Abstract

**Background:**

Fruit and vegetable intake may improve sleep.

**Aims:**

To assess the relationship between fruit and vegetable intake and sleep quality in a longitudinal study.

**Method:**

We analysed longitudinal data from two consecutive population surveys of adults in Agincourt, South Africa (2014–2015 and 2018–2019).

**Results:**

In total, 331 of 2975 participants without poor sleep quality in Wave 1 (11.1%) had incident poor sleep quality in Wave 2, and 270 of 3546 participants who had poor sleep quality in Wave 1 (7.6%) had poor sleep quality in both Waves 1 and 2 (persistent poor sleep quality). The prevalence of poor sleep quality at baseline was 17.2%. In the fully adjusted model for people without poor sleep quality at baseline, higher fruit and vegetable consumption (≥5 servings/day) was positively associated with incident poor sleep quality among men (AOR = 1.43, 95% CI 1.51–2.01) but not among women (AOR = 1.09, 95% CI 0.78–1.46). Two or more servings of fruits were positively associated with incident poor sleep quality among men (AOR = 3.35, 95% CI 1.96–5.72) and among women (AOR = 1.84, 95% CI 1.15–2.94). No models among men and women showed a significant association between vegetable intake and incident poor sleep quality or between fruit and vegetable intake, vegetable intake and persistent poor sleep quality. Fruit intake (one serving) was positively associated with persistent poor sleep quality among men (AOR = 1.76, 95% CI 1.00–3.08) but not among women (AOR = 1.42, 95% CI 0.93–2.18).

**Conclusions:**

Higher fruit and vegetable intake was independently associated with poorer sleep quality among men but not women, and higher fruit but not vegetable intake was associated with poorer sleep quality among both men and women.

Suboptimal sleep is a major public health burden globally: ‘about 25% of adults are dissatisfied with their sleep, 10–15% report symptoms of insomnia associated with daytime consequences, and 6–10% meet criteria for an insomnia disorder’.^1^ For example, in older adults in South Africa, 9.1% reported nocturnal sleep problems^2^ and among older rural South Africans 31.3% of men and 27.2% of women reported nocturnal sleep problems.^3^ It would be important to identify modifiable behaviours, such as the intake of fruit and vegetables, that are beneficial to sleep quality to reduce the public health impact.^4^

A systematic review found that a healthy diet (including fruit, vegetable and milk) was reported to be linked to higher sleep satisfaction.^5^ Several cross-sectional studies, for example among mid-life Mexican women,^6^ young American adults^7^ and older adults in China,^8^ showed that a higher intake of fruit and vegetables was beneficial to sleep quality. Among urban adults in China, higher fruit but not vegetable intake was inversely associated with poor sleep quality.^9^ In Japanese workers, lower vegetable intake increased the odds of poor sleep quality^10^ and among Brazilian workers, inadequate fruit and vegetable intake was associated in both men and women with poor sleep quality.^11^ In another cross-sectional study among university students from 28 countries, higher fruit and vegetable intake decreased the odds of poor sleep quality.^12^

A longitudinal study among young adults in Pennsylvania, USA, found that women with chronic insomnia who increased their intake of fruit and vegetables by three servings/day were twice as likely to report symptoms no longer meeting the threshold for chronic insomnia at 3 months.^4^ It is unclear whether fruit and vegetable intake is associated with incident and persistent poor sleep quality in Africa. Hence, this investigation aimed to evaluate the association between fruit and vegetable intake and incident and persistent poor sleep quality in a longitudinal study in rural South Africa.

## Method

### Participants and procedures

We analysed longitudinal data from two consecutive waves of ‘Health and Aging in Africa: A Longitudinal Study of an INDEPTH Community in South Africa’ (HAALSI). Full information on the sampling methodology has been previously detailed.^13^ The first survey (November 2014 to November 2015) included 5059 individuals (≥40 years of age) and had a response rate of 85.9%;^13^ the second survey (October 2018 to November 2019) included 4176 members of the Wave 1 HAALSI cohort (595 (12%) died during follow-up; 254 (5%) declined participation; 34 (<1%) were not found; response rate: 94%).^14^ The study was conducted by trained field workers in the homes of participants using computer-assisted personal interviewing (CAPI).^13^

The authors assert that all procedures contributing to this work comply with the ethical standards of the relevant national and institutional committees on human experimentation and with the Helsinki Declaration of 1975, as revised in 2008. All procedures involving human participants/patients were approved by the University of the Witwatersrand Human Research Ethics Committee (ref. M141159), the Harvard T.H. Chan School of Public Health, Office of Human Research Administration (ref. C13–1608–02) and the Mpumalanga Provincial Research and Ethics Committee. All participants provided written informed consent.

## Measures

### Outcome variable

In the first and second survey poor sleep quality was assessed using the Brief Version of the Pittsburgh Sleep Quality Index (B-PSQI), which includes five domains: self-reported sleep quality, sleep latency, sleep duration, habitual sleep efficiency and sleep disturbances during the past month.^15^ Summary scores range from 0 to 15 and a B-PSQI cut-off of ≥5 was used to define poor sleep quality; sensitivity and specificity rates are similar to the original PSQI version.^15^

### Exposure variable

Fruit and vegetable intake was identified using the following items:

- ‘In a typical week, on how many days do you eat fruit? (… days)’
- ‘How many servings of fruit do you eat on a typical day? (on any one day) (… servings)’ [use of show cards, one standard serving equals 80 g, 1 medium-size piece of apple, banana, orange, etc.; half a cup of chopped, cooked, canned fruit, etc.; half a cup of fruit juice (juice from fruit, not artificially flavoured)]
- ‘In a typical week, on how many days do you eat vegetables? (… days)’
- ‘How many servings of vegetables do you eat on a typical day? (on any one day) (… servings)’ [use of show cards, one standard serving equals 80 g, 1 cup of raw green leafy vegetables (spinach, salad, etc.), half a cup of tomatoes, carrots, pumpkin, corn, Chinese cabbage, fresh beans, onion, etc., half a cup of vegetable juice].^13^

#### Covariates

- Sociodemographic information, including education, age, marital and migration status, and asset-based household wealth status.^13^
- Current tobacco use, defined as current non-smoking and/or current tobacco smoking.^13^
- Alcohol dependence, assessed using the four-item CAGE questionnaire^16^ (Cronbach's alpha was 0.82).
- Body mass index (BMI), classified according to World Health Organization criteria.^17^
- Hypertension, defined based on Joint National Committee on Prevention, Detection, Evaluation and Treatment of High Blood Pressure criteria.^18^
- Dyslipidaemia, defined as total cholesterol >6.21 mmol/L, HDL-C <1.19 mmol/L, LDL-C >4.1 mmol/L, triglycerides >2.25 mmol/L, or ever diagnosed or medication use for high cholesterol.^13^
- Diabetes, classified as fasting glucose (defined as >8 h) >7 mmol/L (126 mg/dL), or ever diagnosed or medication use for diabetes.^13^
- Physical activity and its levels, classified using the General Physical Activity Questionnaire (GPAQ).^19,20^
- Sedentary behaviour, identified using one question on the GPAQ – ‘time usually spend sitting or reclining on a typical day?’^19^ – and grouped into <4 h, 4 to <8 h and >8 h per day.^21^
- Depressive symptoms, defined as scores ≥3 on the eight-item Center for Epidemiological Studies Depression Scale (CES-D 8)^22^ (Cronbach's alpha 0.66).
- Post-traumatic stress disorder (PTSD) symptoms, defined as ≥4 symptoms identified using a short screening scale for DSM-IV PTSD^23^ (Cronbach's alpha 0.83).

### Data analysis

The proportion of participants with incident and persistent poor sleep quality was calculated and described. The first longitudinal logistic regression analysis excluded those with poor sleep quality at baseline, leaving a sample of 3904 individuals, to estimate incident poor sleep quality (using scores ≥5 as the cut-off), and the second logistic regression analysis estimated longitudinal persistent poor sleep quality (using scores ≥4 as the cut-off). Fruit and vegetable intake was the main predictor, controlled for sociodemographics, substance use, physical activity, sedentary behaviour, BMI and chronic health conditions. Levels of *P* < 0.05 were accepted as statistically significant. Logistic regression models included inverse probability weights that accounted for the probabilities of mortality and attrition during follow-up.^24^ All analyses were performed using Stata SE 15.0 for Windows (College Station, TX, USA).

## Results

### Sample characteristics by incident and persistent poor sleep quality

In total, 331 of 2775 participants without poor sleep quality in Wave 1 (11.1%) had incident poor sleep quality in Wave 2 and 270 of 3546 participants who had poor sleep quality in Wave 1 (7.6%) had poor sleep quality in both Wave 1 and 2 (persistent poor sleep quality). The prevalence of poor sleep quality at baseline was 17.2%. Table 1 shows characteristics of participants by incident and persistent poor sleep quality.

**Table 1:** Sample characteristics by incident and persistent depression, Agincourt, South Africa, 2014–2019

Baseline variables	Sample, *n* (%)	Incident poor sleep quality (scores ≥5 on B-PSQI), %	Persistent poor sleep quality (scores ≥4 on B-PSQI), %
Age, years			
40–49	884 (17.6)	9.5	4.5
50–59	1358 (27.1)	10.2	8.4
60–69	1274 (25.4)	11.3	7.5
70–79	918 (18.3)	13.0	9.7
≥80	583 (11.6)	9.8	8.4
Gender			
Female	2713 (53.6)	10.7	8.7
Male	2346 (46.4)	11.0	6.3
Country of birth			
Mozambique/other	1519 (30.2)	6.9	7.0
South Africa	3508 (69.8)	12.4	7.8
Education			
None	2307 (45.8)	9.3	8.1
1–7 years	1613 (32.0)	11.8	8.2
8–11	537 (10.7)	10.6	5.6
12 or more	585 (11.6)	13.9	6.4
Marital status			
Married/cohabiting	2575 (50.9)	10.7	6.4
Not married	2480 (49.1)	11.0	9.0
Wealth index			
Low	2047 (40.5)	9.1	8.5
Middle	991 (19.6)	12.9	7.0
High	2021 (39.9)	11.6	7.1
Alcohol dependence			
No	4988 (98.7)	10.8	7.6
Yes	68 (1.3)	12.7	4.3
Current tobacco use			
No	4264 (84.4)	10.6	7.6
Yes	790 (15.6)	12.3	7.5
Body mass index			
Normal	1719 (36.7)	11.2	7.6
Underweight	258 (5.5)	8.4	5.9
Overweight	1328 (28.3)	10.3	7.2
Obese	1384 (29.5)	11.5	7.9
Hypertension			
No	2052 (41.6)	11.5	7.7
Yes	2884 (58.4)	10.4	7.4
Diabetes			
No	4093 (88.0)	10.8	7.4
Yes	559 (12.0)	9.3	7.4
Dyslipidaemia			
No	2389 (56.2)	10.9	7.7
Yes	1862 (43.8)	10.9	7.6
Physical activity			
Low	2211 (44.0)	11.4	8.0
Moderate	1143 (22.7)	10.5	7.4
High	1674 (33.3)	10.4	7.5
Sedentary behaviour			
Low	2675 (55.9)	10.4	7.8
Moderate	1632 (34.1)	12.4	7.0
High	475 (9.9)	8.8	10.2
Depressive symptoms			
No	4092 (83.0)	11.0	7.0
Yes	837 (17.0)	12.4	10.9
PTSD symptoms			
No	4697 (95.2)	11.3	7.2
Yes	238 (4.8)	9.5	14.8
Fruit/vegetable consumption, servings/day			
0–1	578 (11.5)	6.4	7.2
2	1404 (28.0)	8.6	7.0
3	1302 (26.0)	11.3	8.6
4	1131 (22.4)	13.7	7.9
5	355 (7.1)	12.0	6.0
6	136 (2.7)	10.2	7.9
7 or more	109 (2.2)	17.3	8.1

B-PSQI, Brief Version of the Pittsburgh Sleep Quality Index; PTSD, post-traumatic stress disorder.

### Correlations between fruit and vegetable intake and incident poor sleep quality

In the fully adjusted model for people without poor sleep quality at baseline, higher fruit and vegetable consumption (≥5 servings/day) was positively associated with incident poor sleep quality among men (AOR = 1.43, 95% CI 1.51–2.01) but not among women (AOR = 1.09, 95% CI 0.78–1.46). Two or more servings of fruits were positively associated with incident poor sleep quality among men (AOR = 3.35, 95% CI 1.96–5.72) and among women (AOR = 1.84, 95% CI 1.15–2.94). No models among men or women showed a significant association between vegetable intake and incident poor sleep quality (Table 2).

**Table 2:** Longitudinal association between fruit and vegetable consumption and incident poor sleep quality (scores ≥5 on B-PSQI), Agincourt, South Africa, 2014–2019^a^

	Unadjusted model	Model 1	Model 2	Model 3	Model 4	Model 4
	
Variable	Odds ratio (95% CI)	Odds ratio (95% CI)	Odds ratio (95% CI)	Odds ratio (95% CI)	Odds ratio (95% CI)	Odds ratio (95% CI)
**Males, 40–59 years of age**						
Fruit/vegetables, servings/day						
0–2	1 (Reference)	1 (Reference)	1 (Reference)	1 (Reference)	1 (Reference)	1 (Reference)
3–4	2.00 (1.40–2.86)***	1.89 (1.32–2.72)***	1.80 (1.28–2.52)***	1.33 (1.01–1.74)*	1.92 (1.27–2.91)**	1.68 (1.10–2.59)*
5 or more	1.92 (1.15–3.18)*	1.84 (1.16–2.92)**	1.57 (0.96–2.55)	1.43 (1.61–2.01)*	1.73 (0.95–3.15)	1.16 (0.50–2.31)
Fruit, servings/day						
0	1 (Reference)	1 (Reference)	1 (Reference)	1 (Reference)	1 (Reference)	1 (Reference)
1	2.26 (1.34–3.91)**	2.10 (1.24–3.57)***	2.28 (1.38–3.77)***	2.22 (1.29–3.82)**	1.82 (1.00–3.31)*	1.91 (1.05–3.47)*
2 or more	3.17 (1.91–5.26)***	2.87 (1.71–4.81)***	3.05 (1.86–5.02)***	3.35 (1.96–5.72)***	3.02 (1.68–5.44)***	1.91 (1.05–3.48)*
Vegetables, servings/day						
0–1	1 (Reference)	1 (Reference)	1 (Reference)	1 (Reference)	1 (Reference)	1 (Reference)
2	0.96 (0.69–1.33)	0.97 (0.70–1.36)	0.99 (0.72–1.36)	0.99 (0.70–1.40)	1.04 (0.71–1.53)	1.31 (0.88–1.96)
3 or more	1.38 (0.82–2.31)	1.52 (0.90–2.57)	1.43 (0.87–2.36)	1.53 (0.88–2.63)	1.57 (0.86–2.90)	1.44 (0.73–2.83)
**Females, ≥60 years of age**						
Fruit/vegetables, servings/day						
0–2	1 (Reference)	1 (Reference)	1 (Reference)	1 (Reference)	1 (Reference)	1 (Reference)
3–4	1.39 (1.00–1.93)*	1.29 (0.95–1.75)	1.29 (0.94–1.77)	1.20 (0.94–1.54)	1.25 (0.85–1.84)	1.36 (0.93–1.97)
5 or more	1.50 (0.93–2.41)	1.43 (0.92–2.21)	1.51 (0.97–2.38)	1.09 (0.78–1.46)	1.32 (0.76–2.30)	1.78 (1.07–2.98)*
Fruit, servings/day						
0	1 (Reference)	1 (Reference)	1 (Reference)	1 (Reference)	1 (Reference)	1 (Reference)
1	1.80 (1.15–2.84)*	1.74 (1.10–2.76)*	1.75 (1.13–2.71)*	1.86 (1.16–2.99)*	1.81 (1.07–3.07)*	1.77 (1.04–3.01)*
2 or more	2.06 (1.33–3.18)***	1.91 (1.22–3.00)**	1.85 (1.20–2.83)**	1.84 (1.15–2.94)*	1.85 (1.09–3.11)*	2.59 (1.54–4.36)***
Vegetables, servings/day						
0–1	1 (Reference)	1 (Reference)	1 (Reference)	1 (Reference)	1 (Reference)	1 (Reference)
2	1.25 (0.81–1.71)	1.23 (0.90–1.70)	1.28 (0.95–1.74)	1.17 (0.84–1.62)	1.17 (0.82–1.68)	0.87 (0.61–1.22)
3 or more	1.02 (0.60–1.75)	1.08 (0.62–1.85)	1.13 (0.68–1.89)	1.12 (0.64–1.95)	0.95 (0.50–1.79)	1.07 (0.60–1.90)

B-PSQI, Brief Version of the Pittsburgh Sleep Quality Index.

a. Model 1: adjusted for age, education, migration, marital and wealth status. Model 2: adjusted for Model 1 variables, plus substance use, physical activity, sedentary behaviour and body mass index. Model 3: adjusted for Model 1 and 2 variables, plus dyslipidaemia, hypertension and diabetes. Model 4: adjusted for Model 1–3 variables plus depressive symptoms and post-traumatic stress disorder symptoms.

*P < 0.05; **P < 0.01; ***P < 0.001.

### Associations between fruit and vegetable intake and persistent poor sleep quality

Table 3 shows, based on the longitudinal analysis, associations between fruit and vegetable intake and persistent poor sleep quality. No models for either gender showed a significant association between fruit and vegetable intake and persistent poor sleep quality. Fruit intake (one serving) was positively associated with persistent poor sleep quality among men (AOR = 1.76, 95% CI 1.00–3.08) but not among women (AOR = 1.42, 95% CI 0.93–2.18).

**Table 3:** Longitudinal association between fruit and vegetable consumption and persistent poor sleep quality (scores ≥4 on B-PSQI), Agincourt, South Africa, 2014–2019^a^

	Unadjusted model	Model 1	Model 2	Model 3	Model 4	Model 4
	
Variable	Odds ratio (95% CI)	Odds ratio (95% CI)	Odds ratio (95% CI)	Odds ratio (95% CI)	Odds ratio (95% CI)	Odds ratio (95% CI)
**Males, 40–59 years of age**						
Fruit/vegetables, servings/day						
0–2	1 (Reference)	1 (Reference)	1 (Reference)	1 (Reference)	1 (Reference)	1 (Reference)
3–4	1.22 (0.86–1.72)	1.27 (0.89–1.81)	1.29 (0.88–1.89)	1.05 (0.70–1.60)	1.19 (0.66–2.17)	1.35 (0.81–2.27)
5 or more	1.05 (0.61–1.82)	1.11 (0.64–1.94)	1.25 (0.71–2.21)	1.41 (0.79–2.54)	1.57 (0.69–3.59)	1.48 (0.70–3.16)
Fruit, servings/day						
0	1 (Reference)	1 (Reference)	1 (Reference)	1 (Reference)	1 (Reference)	1 (Reference)
1	1.31 (0.84–2.05)	1.49 (0.94–2.35)	1.66 (1.00–2.76)	1.76 (1.00–3.08)*	1.24 (0.59–2.61)	1.02 (0.54–1.92)
2 or more	1.26 (0.81–1.96)	1.50 (0.94–2.38)	1.73 (1.03–2.90)*	1.73 (0.97–3.07)	1.25 (0.59–2.63)	1.10 (0.60–2.06)
Vegetables, servings/day						
0–1	1 (Reference)	1 (Reference)	1 (Reference)	1 (Reference)	1 (Reference)	1 (Reference)
2	0.86 (0.62–1.21)	0.83 (0.59–1.17)	0.83 (0.58–1.20)	0.77 (0.52–1.14)	0.75 (0. 44–1.29)	1.19 (0.72–1.98)
3 or more	1.03 (0.59–1.81)	0.90 (0.50–1.62)	1.05 (0.58–1.90)	1.08 (0.57–2.06)	1.28 (0.54–3.02)	2.24 (1.05–4.80)*
**Females, ≥60 years of age**						
Fruit/vegetables, servings/day						
0–2	1 (Reference)	1 (Reference)	1 (Reference)	1 (Reference)	1 (Reference)	1 (Reference)
3–4	1.14 (0.85–1.52)	1.15 (0.86–1.54)	1.17 (0.87–1.60)	1.09 (0.76–1.52)	1.34 (0.88–2.03)	1.41 (0.90–2.22)
5 or more	0.88 (0.55–1.40)	0.83 (0.51–1.34)	0.98 (0.54–1.44)	0.83 (0.49–1.42)	1.01 (0.52–1.97)	1.14 (0.56–2.31)
Fruit, servings/day						
0	1 (Reference)	1 (Reference)	1 (Reference)	1 (Reference)	1 (Reference)	1 (Reference)
1	1.32 (0.91–1.91)	1.40 (0.96–2.04)	1.47 (0.99–2.20)	1.42 (0.93–2.18)	1.52 (0.90–2.59)	1.87 (1.03–3.39)*
2 or more	1.30 (0.91–1.87)	1.38 (0.95–2.01)	1.37 (0.92–2.04)	1.34 (0.88–2.05)	1.43 (0.83–2.44)	1.87 (1.01–3.45)*
Vegetables, servings/day						
0–1	1 (Reference)	1 (Reference)	1 (Reference)	1 (Reference)	1 (Reference)	1 (Reference)
2	0.97 (0.73–1.29)	0.94 (0.70–1.25)	0.99 (0.74–1.36)	0.97 (0.71–1.35)	1.14 (0.79–1.71)	0.96 (0.62–1.48)
3 or more	1.00 (0.63–1.58)	0.89 (0.56–1.44)	0.99 (0.61–1.61)	1.09 (0.65–1.81)	1.36 (0.71–2.60)	0.85 (0.40–1.78)

B-PSQI, Brief Version of the Pittsburgh Sleep Quality Index.

a. Model 1: adjusted for age, education, migration, marital and wealth status. Model 2: adjusted for Model 1 variables, plus substance use, physical activity, sedentary behaviour and body mass index. Model 3: adjusted for Model 1 and 2 variables, plus dyslipidaemia, hypertension and diabetes. Model 4: adjusted for Model 1–3 variables plus depressive symptoms and post-traumatic stress disorder symptoms.

* *P* < 0.05.

## Discussion

In this first longitudinal study on the subject among an ageing population in South Africa, we found that compared with low fruit and vegetable intake, high fruit and vegetable intake was positively associated with incident poor sleep quality 4 years later among men and but not women. Higher fruit intake increased the odds of incident poor sleep quality in both genders. No association was found between vegetable intake and incident and persistent poor sleep quality. Among men only, compared with no serving of fruit, having one fruit serving a day was positively associated with persistent poor sleep quality.

Contrary to these findings, previous cross-sectional and longitudinal studies found an inverse relationship between fruit and vegetable intake, fruit intake and poor sleep quality.^4–6,8,9,11,12^ Similar to the study among Chinese urban adults,^9^ we found no association between vegetable intake and poor sleep quality, whereas in a study in Japan lower vegetable intake increased the odds of poor sleep quality.^10^ It is possible that poor sleep quality increases emotional distress, leading to more fruit consumption, potentially reducing negative mood,^25^ which points to a possible bidirectional relationship between fruit and vegetable consumption and poor sleep quality.^26^

We found gender differences in the positive relationship between fruit and vegetable intake and incident poor sleep quality and fruit intake and persistent poor sleep quality among ageing men in South Africa, whereas among young American adults higher fruit and vegetable intake decreased incident insomnia among women.^4^ In the present study higher fruit intake was strongly associated with higher wealth status (*P* < 0.001, analysis not shown), and higher wealth status may also be associated with high calorie-dense food, which is related to poor sleep quality.^24^ Further investigations are needed to identify possible reasons for the found gender differences in fruit and vegetable intake in relation to poor sleep quality.

### Study limitations

Some data, including sleep quality, were assessed by self-report and not verified by actigraphy or polysomnography, which may have led to an over- or underestimation of poor sleep quality. We were not able to show reasons for poor sleep quality and how this may be related to social or personality characteristics. Furthermore, participants who did not have poor sleep quality in Wave 1 may have had poor sleep quality before. Perceived stress, which might serve as a moderator between fruit intake and poor sleep quality, was not evaluated in this study, and other dietary behaviours that might have contributed to sleep quality, such as calorie-dense food intake, were not measured.

## Data Availability

The data used in this study are publicly available at the Harvard Center for Population and Development Studies (HCPDS) programme website (www.haalsi.org).
